# Reconstruction of the Palmar Defect of the Hand with a Sensory Medial Plantar Artery Flap

**DOI:** 10.3389/fsurg.2022.874629

**Published:** 2022-05-06

**Authors:** Jiayu Li, Xuchao Luo, Yonggen Zou

**Affiliations:** Department of orthopedics, The Affiliated Traditional Chinese Medicine Hospital of Southwest Medical University, LuZhou, China

**Keywords:** sensory medial plantar artery flap, hand wound, palmar defect, reconstruction, microsurgery

## Abstract

**Background:**

There are many approaches to repair the palmar defect of the hand, but due to the complex tissue structure, versatile functions, and high restoration demands, the repair of the palmar defect of the hand has always been a difficult task. Choosing suitable flaps to repair this kind of injury is always a tough challenge for the reconstructive surgeon because of the limitation of the number of arteries in the recipient hand and high restoration demands. The sensory medial plantar artery flap is considered as the ideal choice to repair the palmar defect of the hand. Based on this technique, in the current study, we used the free sensory medial plantar artery flap to reconstruct the palmar defect of the hand and obtained satisfactory results. The purpose of this study was to review the use of the sensory medial plantar artery flap for the reconstruction of the palmar defect of the hand.

**Method:**

From January 2019 to December 2020, nine patients with the palmar defect of the hand underwent extremity reconstruction by a sensory medial plantar artery flap. The indications for this surgery were that the palmar defect of the hand need to be reconstructed and both anterior and posterior tibial arteries should be free-flowing. Patients who had systemic diseases such as diabetes millitus, vascular diseases, heavy smoking histories, and injuries of the donor site were ruled out for the surgery.

**Results:**

Nine patients were successfully treated by using a sensory medial plantar artery flap, with a mean age of 39.44 (range 19–58) years. Five of the patients were male and the remaining four were female. Seven of them had a machine crush, and the other two suffered thermal injury. In all cases, reconstruction was performed during the second stage of treatment. All flaps survived completely, and all of the donor sites healed well in Stage 1, except for one case of ischaemic skin necrosis due to tight sutures, which healed after suture removal.

**Conclusion:**

Our experience showed that the free sensory medial plantar artery flap was an alternative for one-stage reconstruction of the soft-tissue defect in the palm of the hand. This flexible approach not only repaired the soft-tissue defect, but also offered a satisfactory recovery of the sensory of the palm with minimal donor site morbidity.

## Introduction

There are many approaches to repair the palmar defect of the hand, but due to the complex tissue structure, versatile functions, and high restoration demands, the repair of the palmar defect of the hand has always been a difficult task (1). Choosing suitable flaps to repair this kind of injury is always a tough challenge for the reconstructive surgeon because of the limitation of the number of arteries in the recipient hand (2) and high restoration demands. The ALTP (Anterolateral thigh perforator) flap is considered a common option for repairing this injury. However, the ALTP flap cannot meet the high restoration demands of the reconstruction of the palmar defect. The bulky appearance and poor sensory are the disadvantages of adopting the ALTP flap for the reconstruction, which could influence the function and use of the hand (3).

The use of a sensory medial plantar artery flap could be an ideal approach to reconstruct such defect, because it provides a similar tissue structure to the palm of hand, a good sensory, and abundant blood supply (4). The pedicled medial plantar artery flap has been reported for repairing the defect on the heel. SHANAHAN and GINGRASS (5) reported that a pedicled flap is an ideal option for the reconstruction of the heel defect, in terms of both appearance and sensation. Jyoshid and Harsha (6) reported about a free medial plantar artery flap to repair the defect of the great toe pulp. They dissected the flap and then transferred it to the toe to repair the defect. This attempt was successful in repairing the defect of the great toe pulp with the medial plantar artery flap. Based on this technique, in the current study, we used the free sensory medial plantar artery flap to reconstruct the palmar defect of the hand and obtained satisfactory results. The purpose of this study was to review the use of the sensory medial plantar artery flap for the reconstruction of the palmar defect of the hand.

## Methods

From January 2019 to December 2020, nine patients with the palmar defect of the hand underwent extremity reconstruction by the sensory medial plantar artery flap. The indications for this surgery were that the palmar defect of the hand need to be reconstructed and both anterior and posterior tibial arteries should be free-flowing. Patients who had systemic diseases such as diabetes mellitus, vascular diseases, heavy smoking histories, and injuries of the donor site were exempted from the surgery. The age of patients ranged from 19 to 58 years (mean 39.4 years; 5 males and 4 females). Among these patients, 7 had a machine crush, and 2 others had thermal injury. Detailed information about these patients is provided in Table 1. This study was approved by the institutional review committee of our hospital. The protocol was performed in accordance with the ethical standards of the Helsinki Declaration of 1975 and all subsequent revisions. All informed consent was verbally acquired from the patients.

**Table 1:** Patients’ detailed information.

Patient	Sex	Age (years)	Injured hand	Cause of injury	Dimension of the defect (cm^2^)
1	M	19	L	Machine crush	8 × 7
2	F	45	L	Machine crush	4 × 3
3	M	35	R	Thermal injury	7 × 3
4	M	21	R	Machine crush	4 × 2
5	M	58	R	Machine crush	3 × 3
6	F	54	L	Machine crush	5 × 6
7	M	53	R	Thermal injury	4 × 3
8	F	44	R	Machine crush	8.5 × 3.5
9	F	26	R	Machine crush	6 × 3
Average		39.44			
STDEV		14.79			

*M, Male; F, Female; L, Left; R, Right.*

### Technique

The flap was designed on the basis of the palmar defect of the hand. The donor site was covered by skin grafting, and if the donor site was large, a free abdominal flap was used for the repair.

### Operative Techniques

All patients underwent emergency debridement after admission, and the bone fracture of hand was treated with K-wire fixation. All flap surgeries were performed during the second stage. Before the flap surgery, the Doppler probe was used to detect the locations of perforator vessels of the medial plantar artery, and then the locations were marked on the skin. General anaesthetic was used in all cases. The tourniquets were placed on the leg and the upper arm. The artery, vein, and nerve that were used for the anastomosis were detected in the recipient site, and the defects of nerve and tendon were repaired at the same stage.

Briefly, perforators were marked on the skin of the donor site preoperatively. The flap was designed on the basis of 5% enlargement of the defect size and carried 1 to 2 perforators depending on the size of the flap. The skin was incised at the pulsation point of the posterior tibial artery exiting the ankle canal to reveal the bifurcation of the medial and lateral plantar arteries from the posterior tibial artery and to find the medial plantar artery. A distal flap incision was made on the proximal side of the first metatarsal head and down to the deep fascia. The main trunk of the medial plantar artery and the accompanying veins and nerves were detected within the hallux muscle and flexor digitorum brevis. The perforators were carefully detected and traced back to the main trunk of the medial plantar artery. The vessels were dissected to the proximal posterior tibial artery and vein for a sufficient length and the corresponding sensory nerves of the flap were carried. After the surrounding tissues were dissected, the communication of perforators between the skin and the main trunk of the medial plantar artery was reserved. If the cutaneous branch of the medial plantar artery is thicker than the other, it needs to be ligated distally (7).

After the flap was transferred to the injured hand, the distal edge of the flap was sutured on the recipient hand. The microsurgical anastomosis of the artery, vein, and nerve was done to the ulnar artery or common palmar digital artery, vein, and nerve in the recipient site, respectively. The edge of the flap was loosely sutured. The donor site was closed directly or with skin grafting, except in one case, in which the wound of the donor site was covered by the SCIA (Superficial Iliac Circumflex Artery) flap due to its complex shape.

### Evaluation of Outcomes

A group of senior hand surgeons performed the assessments. The Michigan Hand Questionnaire (MHQ) was used to evaluate the subjective function (except pain scoring, range: 0–100) and pain scoring (range: 0–100). The Vancouver Scar Scale was used to assess the scar quality of the donor site.

## Results

Nine patients were successfully treated by using the sensory medial plantar artery flap with a mean age of 39.44 (range 19–58) years. Five of the patients were male and the remaining four were female. Seven of them had a machine crush, and the other two suffered thermal injury. In all cases, reconstruction was performed during the second stage of treatment. All flaps survived completely, and all of the donor sites healed well in Stage 1, except for one case of ischaemic skin necrosis due to tight sutures, which healed after suture removal.

In total, the sizes of the flap ranged from 4 × 2 cm to 8 × 7 cm. The total surgery time varied from 5.0 to 11.0 h with an average time of 7.14 h. The MHQ score of the function was 34.54 ± 3.0 points, and the pain score was 34.44 ± 20.53 points. Primary closure was performed in three cases, skin grafting was used in five cases, and one donor site was covered by an SCIA flap, due to its complex shape. The scar scoring was from 3 to 8 with an average of 4.6. The sensation in the flaps was S3. The mean follow-up time was 20.44 months (ranging from 8 to 30) (Table 2). All flaps showed a satisfactory recovery of sensory (all patients could lead a normal life), and there was a slight diminishment of the medial plantar sensation, with no ulcer production. No significant effect on walking function was noticed.

**Table 2:** Surgical outcomes.

Patients	Location of the flap	Area of the flap (cm^2^)	Operation time (hours)	Flap survival	Treatment of donor site	MHQ Score	Pain score	Vancouver Scar Scale	Follow-up time (months)
1	R	10 × 8	11.0	Complete	F	33.6	40	3	24
2	L	5 × 4	6.0	Complete	S	34.2	40	4	12
3	R	8 × 4	7.5	Complete	S	30.1	45	3	18
4	L	5 × 3	5.5	Complete	P	39.3	60	5	16
5	L	4 × 4	5.0	Complete	P	30.4	0	6	28
6	R	6 × 7	6.25	Complete	S	35.2	0	4	8
7	R	5 × 4	8.0	Complete	S	36.7	45	8	24
8	R	9.5 × 4.5	8.5	Complete	S	34.7	40	3	30
9	R	7 × 4	6.5	Complete	P	36.7	40	5	24
Average			7.14			34.54	34.44	4.6	20.44
STDEV			1.9			3.0	20.53	1.7	

*L, Left; R, Right; F, Flap; P, Primary closure; S, Skin grafting; MHQ, Michigan Hand outcome Questionnaire.*

**Figure 1:**
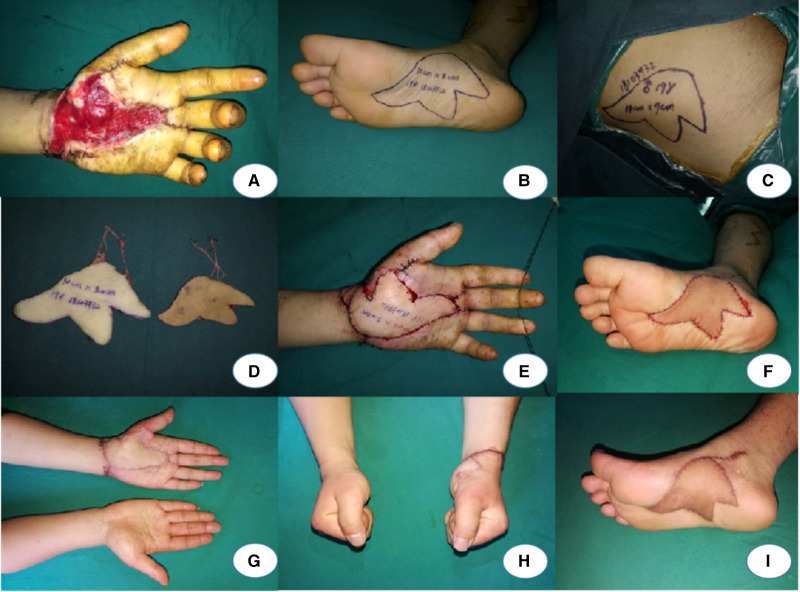
**A**: Palmar soft-tissue defect on the left hand. **B–D**: Design of the flaps. **E**, **F**: The defect of palm was repaired by medial plantar artery flap, and a SCIP was used to cover the donor site. **G–I**: A 24-month follow-up after the operation.

A 19-year-old male sustained a machine crush and presented with a palmar soft-tissue defect on his left hand (Figure 1A). The VSD (vacuum sealing drainage) was used after the emergency debridement. The defect was covered during the second stage of treatment. The flap was designed according to the shape of the defect (Figure 1B,C). Before the harvest of the flap, a group of surgeons discussed the treatment of the donor site. Because of the young age of the patient and the irregularity of the wound which could not be directly closed, an SCIA flap (Figure 1D) was used to replace the skin grafting in order to reduce the damage to the donor site and the impact on the walking function. The size of the sensory medial plantar artery flap was 10*8 cm, and the size of the SCIA flap was 11*9 cm. The artery and vein of the flap were anastomosed to the radial artery and vein in the recipient site, respectively (Figure 1E). The donor site on the foot was covered by the SCIA flap (Figure 1F), and the donor site of the SCIA flap was closed directly. The postoperative course was an uneventful 7 days after operation. The patient received a 24-month follow-up post operation, and the appearance of the flaps showed a satisfactory contour (Figure 1G). There was no excessive bulk and the patient could accomplish grasping and writing functions well (Figure 1H,I). The MHQ score of the hand function was 33.6 points. The pain score was 40. The sensation in the flaps was S3. The scar scoring was 3 points. All the donor sites healed well in Stage 1 (Table 2).

**Figure 2:**
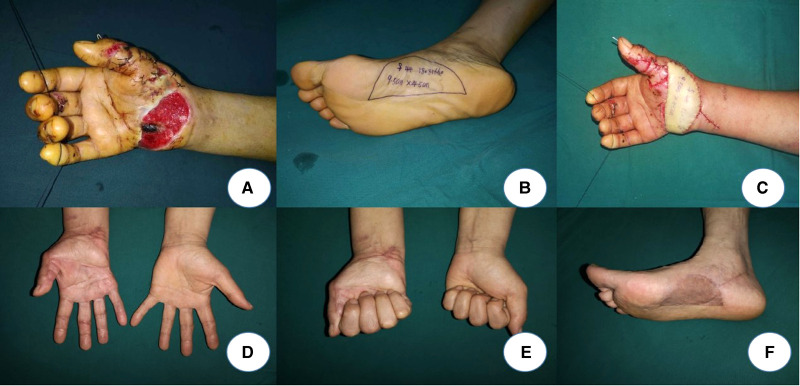
**A**: Soft-tissue defect on the right hand. **B**: Design of the flap. **C**: Coverage of the flap. **D–F**: A 30-month follow-up after the operation.

A 44-year-old female was injured in a machine crush that caused a large soft-tissue defect on her right hand. After the emergency debridement, the tendons and bones were exposed. The VSD was used after the emergency debridement until the tendons and bones were almost covered by the granulation tissue (Figure 2A). To reconstruct the defect and maintain the sensory of the palmar as normal as possible, a sensory medial plantar artery flap was designed to cover the injury in Stage 1. The flap was harvested measuring 9.5 cm × 4.5 cm (Figure 2B,C). The donor area was closed with skin grafting. The postoperative course was uneventful. The flap survived uneventfully. The patient had full functional recovery with satisfactory appearance and sensory restoration after 30 months’ follow-up (Figure 2D–F).

## Discussion

The palm of the hand differs (8) from other parts of the skin, in that there is a thicker cuticle layer on the surface, a thicker fat pad under the skin, and many vertical dermal ligaments connecting the skin to the aponeuroses, making it less likely to slide, thus facilitating the hand to pinch and hold things and resist pressure. The papillae of the palm are rich in sensory nerve endings and receptors, so the senses are very sensitive. The soft tissue injury in the hands would cause hand function loss. For example, the exposure of deep tissues in the palm would influence the function of griping, grasping, and holding profoundly. Because the toughness of the skin and the thickness of the cuticle are not appropriate to the palm, and skin grafting can cause scar contracture, poor sensory, and poor wear resistance (9), grafting is not a suitable method to cover deep wounds in the palm. Various flaps are commonly used to reconstruct the palmar defect of the hand in clinical practice. The pedicled abdominal flap (10) and the forearm retrograde island flap (11) are commonly used to repair hand wounds. The advantages are that such procedures are easy to perform, do not require anastomosis, and their operating time is short, but the greatest disadvantage is that they cannot carry sensory nerves to reestablish sensation in the skin of the recipient area, which can lead to the development of ulcers. Free flaps such as the ALTP (Anterolateral Thigh Perforator) flap can carry the nerve to restore the sensory, but their bulky appearance, significant color difference, and slippage can lead to profound functional limitations of the hand.

Alternatively, the use of the sensory medial plantar artery flap for reconstruction is advantageous, as both the hand and the foot originate in the mesoderm and ectoderm (12). So, the palm and sole are similar in many ways. The skin structure of the medial plantar aspect of the foot is most similar to that of the palm, with constant vascular anatomy and a thick caliber (13) that facilitate anastomosis. The flap is rich in sensory nerves and after long-term follow-up, the flap’s texture, thickness, toughness, and palmar pattern are very similar to the surrounding skin of the recipient site, with very few ulcers and good function of the affected limb. The medial plantar area is a non-weight-bearing area, and the harvest of the flap causes no damage to the blood supply and standing or walking functions of the foot. This is why the medial plantar flap is regarded as the ideal flap for the repair of palmar defects. The medial plantar artery is a direct continuation of the medial plantar artery and runs along the medial edge of the plantar fascia with 2–4 cutaneous branches innervating the non-weight-bearing region of the plantar area, forming the source of blood supply to the flap. The superficial branch has a mean length of 40.8 mm and an outer diameter of 1.0 mm; the deep branch is a direct continuation of the medial plantar artery and runs along the deep anterior aspect of the lesser adductor muscle, with a mean length of 67.8 mm and an outer diameter of 1.1 mm.

The advantages of this flap are as follows: ① The reason for the resistance to wear is not only the specific anatomy (a thicker fat pad under the skin and many vertical dermal ligaments connecting the skin to the aponeuroses, making it less likely to slide), but also the sensory innervation, where the nerves feel the wear, compensatingly leading to a thickening of the keratin and achieving adaptive resistance. ② The skin pattern of the medial plantar skin is similar to that of the palm of the hand, and the appearance of the flap after transplantation is satisfactory. ③ The flap has constant vascularity, a thicker diameter, a high patency rate after anastomosis, rapid blood flow, is less prone to embolism, a thicker diameter of the accompanying veins, and is less prone to venous crisis. ④ The flap is taken from the medial area of the foot, the donor site is hidden, and it is a non-weight-bearing area of the foot, which does not affect the appearance or function of the foot. The disadvantages are as follows: ① There is only a small area for flap excision. ② After flap excision, the donor site cannot be closed directly and needs to be covered with a skin graft or a free flap from another site.

Precautions should be taken when harvesting the flap to avoid inappropriate irritation of the vessels leading to spasm, which may affect the blood supply to the flap; intraoperative removal of the flap can be carried out with the metatarsal tendon and deep fascia to better protect the vascular tip. The restoration of sensation to the palm of the hand is essential, and it is, therefore, important to carry the nerve with the donor nerve suture during excision. Of course, care must be taken to protect the muscular branches of the medial plantar nerve and the sensory branches of the toes, as this can lead to ulceration and impaired walking. The donor area is in the foot and the flap is suitable for repairing small-to-medium-sized soft tissue defects, but if the defect is too large, some degree of damage to the weight-bearing area of the foot may occur.

## Conclusion

Our experience in this study showed that the free sensory medial plantar artery flap was an alternative for one-stage reconstruction of the soft-tissue defect in the palm of the hand. This flexible approach not only repaired the soft-tissue defect, but also offered a satisfactory recovery of the sensory of the palm with minimal donor site morbidity.

## Data Availability

The original contributions presented in the study are included in the article/Supplementary Material; further inquiries can be directed to the corresponding author/s.
